# Differentiating TP53 Mutation Status in Pancreatic Ductal Adenocarcinoma Using Multiparametric MRI-Derived Radiomics

**DOI:** 10.3389/fonc.2021.632130

**Published:** 2021-05-17

**Authors:** Jing Gao, Xiahan Chen, Xudong Li, Fei Miao, Weihuan Fang, Biao Li, Xiaohua Qian, Xiaozhu Lin

**Affiliations:** ^1^ Department of Nuclear Medicine, Ruijin Hospital, School of Medicine, Shanghai Jiao Tong University, Shanghai, China; ^2^ School of Biomedical Engineering, Shanghai Jiao Tong University, Shanghai, China; ^3^ Department of Nuclear Medicine, Qingdao Municipal Hospital, Qingdao, China; ^4^ Department of Radiology, Ruijin Hospital, School of Medicine, Shanghai Jiao Tong University, Shanghai, China; ^5^ Department of Radiology, Ruijin Hospital North, Shanghai, China

**Keywords:** pancreatic ductal adenocarcinoma, TP53, radiomics, support vector machine, multiparametric MRI

## Abstract

**Objectives:**

This study assessed the preoperative prediction of TP53 status based on multiparametric magnetic resonance imaging (mpMRI) radiomics extracted from two-dimensional (2D) and 3D images.

**Methods:**

57 patients with pancreatic cancer who underwent preoperative MRI were included. The diagnosis and TP53 gene test were based on resections. Of the 57 patients included 37 mutated TP53 genes and the remaining 20 had wild-type TP53 genes. Two radiologists performed manual tumour segmentation on seven different MRI image acquisition sequences per patient, including multi-phase [pre-contrast, late arterial phase (ap), portal venous phase, and delayed phase] dynamic contrast enhanced (DCE) T1-weighted imaging, T2-weighted imaging (T2WI), Diffusion-weighted imaging (DWI), and apparent diffusion coefficient (ADC). PyRadiomics-package was used to generate 558 two-dimensional (2D) and 994 three-dimensional (3D) image features. Models were constructed by support vector machine (SVM) for differentiating TP53 status and DX score method were used for feature selection. The evaluation of the model performance included area under the curve (AUC), accuracy, calibration curves, and decision curve analysis.

**Results:**

The 3D ADC-ap-DWI-T2WI model with 11 selected features yielded the best performance for differentiating TP53 status, with accuracy = 0.91 and AUC = 0.96. The model showed the good calibration. The decision curve analysis indicated that the radiomics model had clinical utility.

**Conclusions:**

A non-invasive and quantitative mpMRI-based radiomics model can accurately predict TP53 mutation status in pancreatic cancer patients and contribute to the precision treatment.

## Introduction

Pancreatic ductal adenocarcinoma (PDAC) is characterized by late diagnosis, high mortality rate, and low overall survival (1). The poor prognosis and inefficiency of current treatments are primarily caused by chemoresistance for PDAC. The absence of key genetic alterations is the main driver of chemoresistance, which can disorder the apoptotic (2). As one of the major genetic mutations,TP53 mutations were seen in 70% of pancreatic cancer (3). TP53 encodes the p53 protein, as a tumour suppressor gene, which restricts cell proliferation in many cellular processes, involved in DNA repair, cell-cycle arrest, and apoptosis (4). These mutations are linked to poor patient prognosis (5), although literature regarding its influence is controversial. In addition, resistance to some therapeutic modalities, such as gemcitabine and 5-fluorouracil-based therapies (6, 7). However, novel regimens that target pancreatic cancer cells are emerging. TP53 may be an attractive target for gene augmentation therapy in pancreatic cancer (8).

Unlike surgery resection or biopsy, radiomics as a non-invasive tool was used to detect TP53 mutations (9), which can assess tumour heterogeneity by evaluating the grey-level intensity of pixels and their position in a medical image (10). Currently, CT-based radiomics have been widely used for predicting gene expression and survival prediction (11, 12) in PDAC patients. Some studies use MRI texture recognizing the status of TP53 in many cancers (13, 14). Standard methods of texture analysis methods are 2D or 3D approaches. 3D imaging features may capture tissue properties of the entire tissue more accurately, which improves the predictive power of imaging biomarkers in pancreatic cancer. However, 3D whole-tumour analysis is complicated and time-consuming. Although 2D or 3D texture analysis (TA) has been previously used to extract CT/MRI image features to predict gene status (15), to our best knowledge, neither 2D or 3D TA of mpMRI has been aimed at predicting TP53 status before.

In this study, support vector machine (SVM) radiomics models were constructed using 2D and 3D texture features extracted from mpMRI for the assessment of pre-operative TP53 mutation in PDAC patients.

## Materials and Methods

### Study Population and Tissue Samples

Following local Institutional Review Board approval, this retrospective study was approved with a waiver to obtain written informed consent. Patients with pancreatic ductal adenocarcinoma (PDAC) treated with surgery-based strategy at Ruijin Hospital from January 2016 to December 2016 were included in this study. The inclusion criteria were as follows: 1) pathologic confirmation of PDAC; 2) available the next-generation sequencing(NGS)-based TP53 sequence analysis; 3) MR images contained all of the following sequences: multi-phase [pre-contrast, late arterial phase (ap), portal venous phase (pp), and delayed phase (dp)] dynamic contrast enhanced (DCE) T1-weighted imaging (T1WI, T1), T2-weighted imaging (T2WI, T2), DWI and ADC.

Tissue samples from surgical resections of all 57 PDAC patients were analyzed. Genomic DNA was extracted from formalin-fixed paraffin-embedded (FFPE) tissue, and TP53 mutations were examined using NGS approach.

### Image Acquisition

MR images were acquired on 1.5 T MRI scanners (N=9) or 3.0 T MRI scanners (N=48). The MRI examination included different acquisition sequences, including axial turbo spin-echo T2 sequence with fat saturation, DWI using a single-shot echo-planar imaging pulse sequence with b-values (0, 600 or 800 s/mm^2^), pre- and post-contrast fat-suppressed T1-weighted gradient sequences with intravenous administration of gadopentetate dimeglumine-diethylenetriaminepentaacetic acid (Gd-DTPA) contrast. The apparent diffusion coefficient was calculated by using a monoexponential function with b-values of 0 s/mm^2^ and 600 or 800 s/mm^2^. Gd-DPTA dose of 0.1 mmol per kg and a flow rate of 2 mL per sec were achieved. Late arterial phase, portal vein phase and delayed phase were acquired approximately 35-, 60-, and 90- seconds after contrast injection. Scan parameters for the MRI sequences are summarized in the **Supplementary Table 1**.

### Segmentation of Region of Interest (ROI)

ROIs of the tumour were manually segmented by a junior radiologist in ITK‐SNAP software [Version 3.6 (16)] and were validated by an experienced senior radiologist. ROIs were manually drawn along the margin of the tumor covering the largest possible region. ROIs were delineated on multi-phase (pre-contrast, ap, pp, dp) dynamic contrast enhanced T1WI, T2WI, DWI and ADC images.

### MRI Image Feature Extraction

A total number of 558 2D image features from the largest cross-sectional area of a tumor and 994 3D image features were extracted from the entire tumor area for each image. In order to improve multiparametric MRI radiomic feature robustness, the image intensity of each sequence was normalized to the range of 0–1. MRI voxel was resampled to 1 mm× 1 mm× 1 mm to reduce the variability of different scanners. The features included first-order features, shape features, gray level co-occurrence matrix (GLCM) features, gray level dependence matrix (GLDM) features, gray level run length matrix (GLRLM) features, gray level size zone matrix (GLSZM) features and neighbouring gray-tone difference matrix (NGTDM) features of original images, wavelet transformed images and gradient images. The specific number of features are listed in **Supplementary Table 2**. The feature extraction procedure was implemented in the Pyradiomics package (python 3.6) (17).

### Feature Selection and SVM Model Construction

In total, 378 radiomics models (i.e. 126×3 = 378) were formed based on 2D,3D, and 2D/3D combination from the seven different mpMRI datasets. For each model, feature selection and TP53 gene prediction were performed separately.

For 2D features, the number of models can be calculated by :

$$number=C_{7}^{1}+C_{7}^{2}+C_{7}^{3}+C_{7}^{4}+C_{7}^{5}+C_{7}^{6}+C_{7}^{7}=126$$

Where

$$C_{n}^{m}=\frac{n!}{m!(n-m)!}$$

Among the extracted features, some were highly correlated and some had poor ability to assess TP53 gene mutation. Besides, it’s unknown which model features play the main role. Therefore, in order to remove the most redundant and irrelevant features and choose the most important and typical features, we performed feature selection base on DX score method (18) before gene prediction. DX score is an effective method to measure the difference between positive and negative samples (19). The higher the score is, the stronger the discriminating ability to distinguish between two types of samples. It can be mathematically defined as:

(1) $$D(X)=\frac{{\left(m_{positive}-m_{negative}\right)}^{2}}{d_{positive}^{2}+d_{negative}^{2}}$$

where *m_positive_* and *m_negative_* are the mean value respectively, *d_positive_* and *d_negative_* are the standard deviation of the feature X.

(2) $$m=\frac{1}{N}\sum_{i=1}^{N}x_{i}$$

(3) $$d=\sqrt{\frac{1}{N-1}\sum_{i=1}^{N}{\left(x_{i}-m\right)}^{2}}$$

where x is the value of the feature X with respect to positive (or negative) samples, N is the total number of positive (or negative) samples.

Then, features were ranked from the most important to the least important. The top n(1≤n≤N) features were input to the SVM classifier model of the initial parameter in turn. We assessed its classification performance using the accuracy rate, so the number of features in the feature set is the abscissa (X-axis), and the classification accuracy of each feature set is plotted on the ordinate (Y-axis). The feature set with the highest accuracy was selected.

Next, the SVM model was constructed using the features selected above. A grid search was conducted on the trade-off coefficient (C) and the kernel function parameter (gamma). Then, features were selected again using the optimal SVM model. The SVM package LIBSVM (20) was used for SVM model construction due to its well-known performance. Five-fold cross-validation was applied to evaluate model performance during the experiment to avoid over-fitting.

### Statistical Analysis

Independent sample t tests and chi-squared test were used to compare continuous variables and categorical variables, respectively. Receiver operating characteristic (ROC) curve, calibration curve, and decision curve analysis (DCA) were used to evaluate and choose between the generated models. The corresponding values of the area under the ROC curve (AUC) and the Brier score (BS) were also calculated. The higher the AUC value, the better the model performance. On the other hand, the Brier score is the measure of the calibration curve. For a set of prediction values, the lower the Brier score, the better the prediction calibration. BS score is mathematically calculated as:

(4) $$\text{BS}=\frac{1}{4}\sum_{t=1}^{N}{\left(p_{t}-o_{t}\right)}^{2}$$

where *p_t_* is the probability of prediction, *o_t_* is the real probability of sample t and N is the number of samples. The calibration curve and Brier score were obtained with Sklearn (python 3.6). A t-test was conducted to evaluate significant differences between the two models. Decision curve analysis was used to quantify the net benefit of models to guide subsequent actions (21). The ROC and AUC were used to evaluate the diagnostic performance of radiomics models. The ROCs of radiomics models were compared using the DeLong test (22) implemented in MATLAB R2018a(Mathworks, Natick, MA, USA). A p value < 0.05 was considered statistically different.

## Results

### Patients

Out of 57 patients, 37 had mutated TP53 genes, and 20 had wild-type TP53 genes. The characteristics of patients are summarized in **Table 1**. There were no statistical differences in age (p = 0.770), gender (p = 0.397), histologic stage (p = 0.402) between the TP53 mutation and TP53 wild-type groups.

**Table 1:** Clinical and pathological analysis of patients with or without TP53 mutation.

Characteristic	Wild-type TP53 (N=20)	Mutated TP53 (N=37)	P-value
Mean age(y)	60	62	0.770
Gender			0.397
Female	9	12	
Male	11	25	
Grade			0.402
1	4	3	
2	11	25	
3	5	9	

### Radiomics Model Performance and Feature Selection

**Figure 1:**
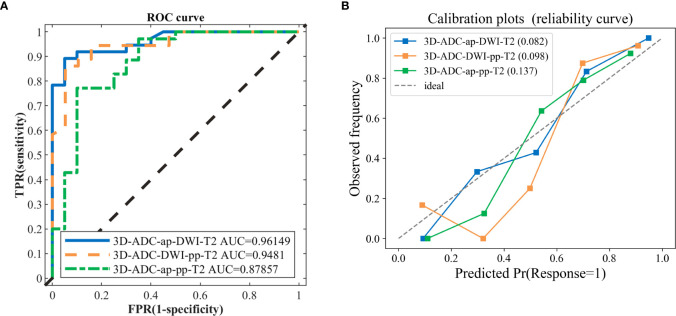
The ROC curves and calibration curves of three classification models. **(A)** the 3D-ADC-ap-DWI-T2 model (best one, AUC=0.9615), 3D-ADC-DWI-pp-T2 model (AUC=0.9481) and 3D-ADC-ap-pp-T2 model including the fewest features model (AUC=0.8786). **(B)** Observed (y-axis) versus the predicted probability frequency (x-axis). The closer the points appear along the main diagonal, the better calibrated. 3D-ADC-ap-DWI-T2 is the closet to the diagonal dotted line, which represents perfect calibration.

In total, 378 radiomics models were formed based on the combination of 7 sequences in the 2D, 3D, and 2D/3D combination. The model including ADC, ap (DCE T1WI late arterial phase), dp (DCE T1WI delayed phase), DWI, pp (DCE T1WI portal venous phase), T2 (T2 weighted imaging) sequence (ADC_ap_dp_DWI_pp_T2) generated the best performance with an AUC of 98.02%, including 41 features. Models with fewer predictors and similar predictive performance from all combinations of modalities were also selected, to avoid over-fitting and redundancy. Comparing the AUCs from the above-mentioned model presenting the highest value of AUC revealed that there were seven models with no statistically significant difference (P>0.05) in their abilities to differentiate (**Table 2**). The models with the top two AUC values and the least number of features were selected and named as the model I, II, and III. Details of extracted radiomic features in the three model was listed in the **Table 3**. The overall ability of model I, model II, and model III for identifying TP53 status are represented by their AUC values (96.15,94.81,87.85%, respectively; **Figure 1A**). Furthermore, **Figure 1B** shows the Brier score, measuring overall model performance *via* mean squared error between predicted probabilities and expected values.

**Table 2:** Comparison of Performance of the models between the highest one versus the other models with fewer predictors.

Dimension	Model Selection	ACC	AUC	Feature Numbers	P-value
3D	ADC_ap_dp_DWI_pp_T2	0.919	0.980	41	
**3D**	**ADC_ap_pp_T2**	**0.836**	**0.879**	**5**	**0.0501**
3D	ADC_dp_pp_T1_T2	0.860	0.807	5	0.0195
2D_3D	ADC_dp_pp_T1_T2	0.860	0.808	5	0.0174
3D	ap_dp_DWI_pp_T1	0.781	0.773	6	0.0085
3D	dp_DWI_pp_T1_T2	0.855	0.803	7	0.0124
3D	ap_dp_DWI_pp_T1_T2	0.855	0.817	7	0.0150
**3D**	**ADC_ap_pp_T1_T2**	**0.851**	**0.866**	**11**	**0.0673**
**3D**	**ADC_ap_dp_pp_T2**	**0.877**	**0.915**	**11**	**0.1148**
2D_3D	ap_dp_DWI_pp_T1	0.800	0.795	11	0.0152
**2D_3D**	**ap_dp_pp_T1_T2**	**0.894**	**0.888**	**11**	**0.1066**
**3D**	**ADC_ap_DWI_pp_T2**	**0.877**	**0.931**	**12**	**0.1648**
**3D**	**ADC_ap_DWI_T2**	**0.915**	**0.962**	**11**	**0.9410**
3D	ADC_dp_T1_T2	0.855	0.817	11	0.0145
**3D**	**ADC_DWI_pp_T2**	**0.891**	**0.948**	**11**	**0.5946**

Models similar to the optimal model (P value> 0.05) are highlighted by bold text; ACC, Accuracy; AUC, area under receiver operating characteristic (ROC) curve.

**Table 3:** Details of extracted radiomics features in the three models.

Models	Feature Names	Number of features
3D_ADC_ap_DWI_T2 (Model I)	ADC-wavelet-LLL_firstorder_Maximum ADC-original_firstorder_Maximum ADC-gradient_glcm_MCC ADC-original_shape_Sphericity ap-wavelet-HHL_ngtdm_Busyness ap-gradient_glcm_Correlation DWI-wavelet-LHH_glszm_SmallAreaEmphasis T2-wavelet-HLH_glcm_Correlation T2-wavelet-HHL_firstorder_Mean T2-wavelet-HLH_glcm_MCC T2-wavelet-HHL_firstorder_Median	11
3D_ADC_DWI_pp_T2 (Model II)	ADC-wavelet-LLL_firstorder_Maximum ADC-gradient_glcm_MCC ADC-original_firstorder_Maximum ADC-gradient_ngtdm_Busyness DWI-wavelet-LHH_glszm_SmallAreaEmphasis pp-wavelet-HHL_glrlm_ShortRunEmphasis pp-wavelet-HLL_glcm_Idn T2-wavelet-HLH_glcm_Correlation T2-wavelet-HHL_firstorder_Median T2-wavelet-HLH_glcm_MCC T2-wavelet-HHL_firstorder_Mean	11
3D_ADC_ap_pp_T2 (Model III)	ADC-wavelet-LLL_firstorder_Maximum ap-gradient_glcm_Correlation ap-wavelet-LLL_glcm_MCC T2-wavelet-HHL_firstorder_Median T2-wavelet-HLH_glcm_Correlation	5

**Figure 2:**
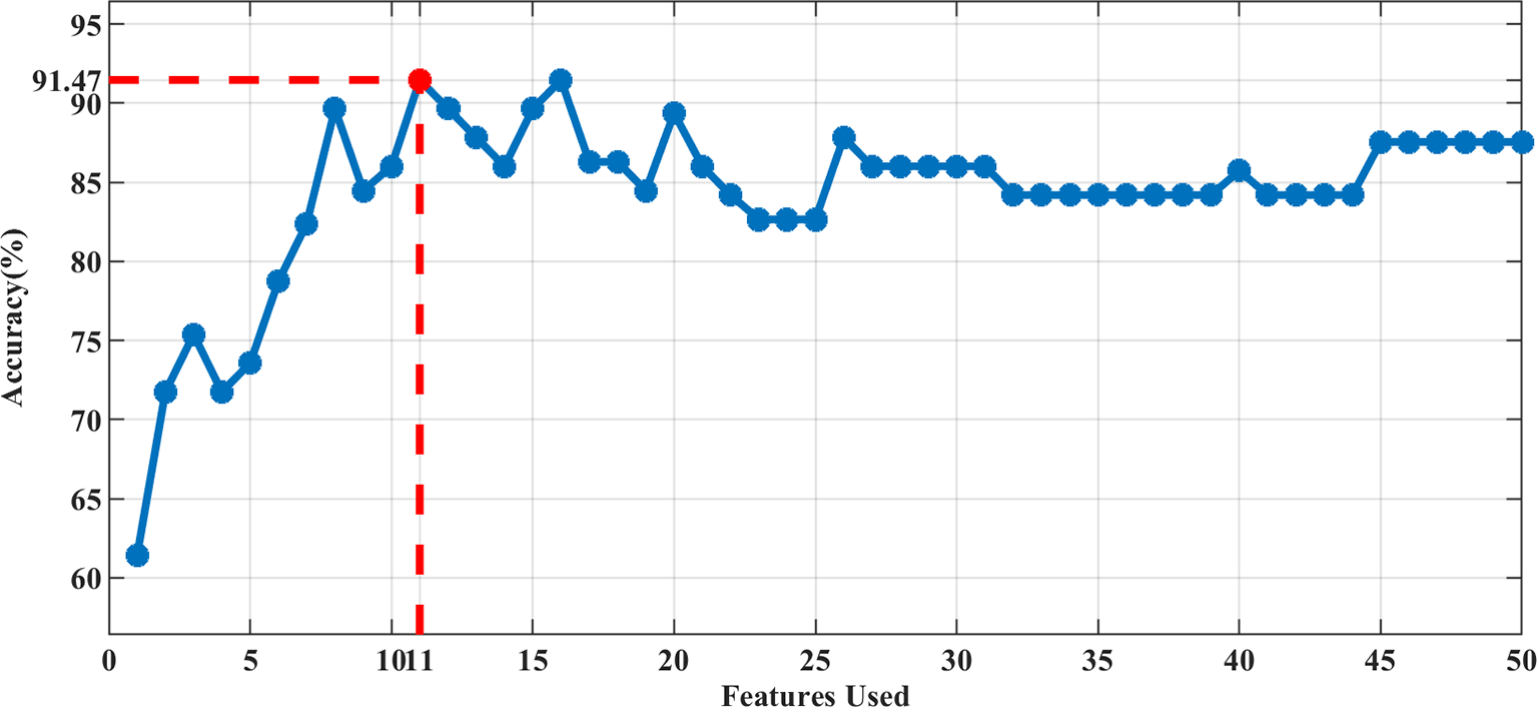
The accuracy of 5-fold CV by adding features sequentially. The best performance was achieved using the Top-11 feature set in the 3D-ADC-ap-DWI-T2 multiparametric model.

In our study, the 3D-ADC-ap-DWI-T2 model had the highest AUC and the lowest Brier score, indicating superior model performance. The optimal feature subset selected by DX score based on SVM classifier accuracy (using 5-fold CV accuracy), included the top-11 feature set for predicting TP53. **Figure 2** illustrates the feature selection process for the best model (3D-ADC-ap-DWI-T2).

### Clinical Usefulness

**Figure 3:**
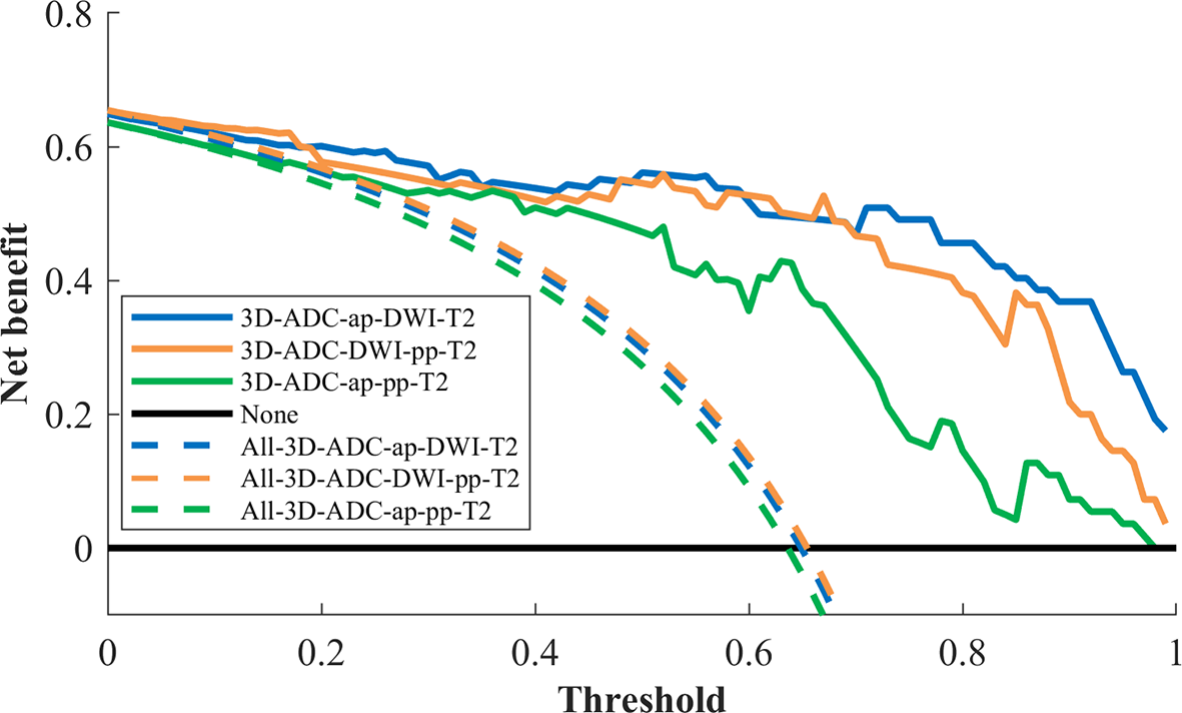
Decision curve analysis for 3 radiomics models. The y-axis measures the net benefit. The x-axis represented the threshold probability. The dashed line represents the assumption that all patients underwent model I, model II and model III test; the horizontal black line represents the assumption that no patients underwent MRI test; The blue line represents the 3D-ADC-ap-DWI-T2 model; the orange line represents the 3D-ADC-DWI-pp-T2 model; the green line represents the 3D-ADC-ap-pp-T2 model.

**Figure 4:**
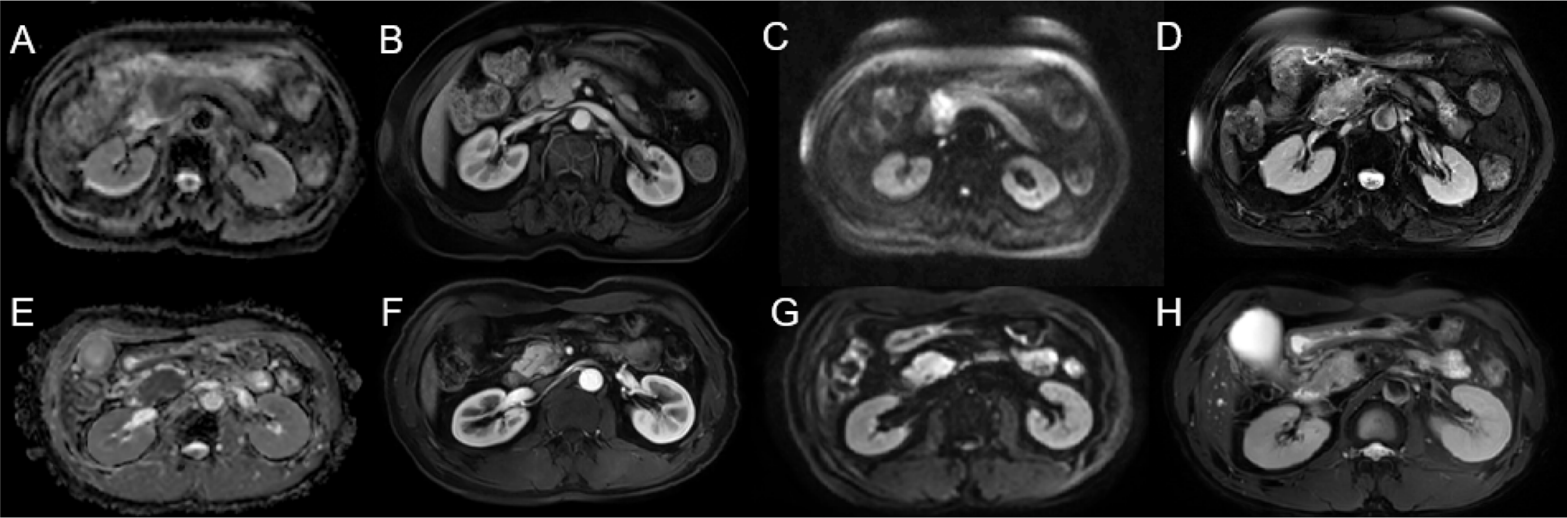
Two cases of multiparametric MRI images from pancreatic ductal adenocarcinoma patients with wild-TP53 **(A–D)** from a 78 year-old women and TP53 mutation **(E–H)** from a 53 year-old men. ADC map showed hypointense lesion **(A, E)**. Slightly hypovascular lesion on late arterial phase **(B, F)**. DWI sequence showing hyperintense lesion **(C, G)**. T2WI showed slight hyperintensity of the pancreatic head mass **(D, H)**.

Decision curve analysis was used to assess the clinical usefulness of the radiomics models to guide identifying TP53 status (**Figure 3**). Three radiomics models had clinical utility due to the net benefit of models were greater than treating all and none patients (**Figure 4**). **Figure 4** showed two cases of multiparametric MRI images from pancreatic ductal adenocarcinoma patients with wild-TP53(A-D) and TP53 mutation(E-H). MRI findings were similar, and gene mutation could not be distinguished. However, gene mutations can be accurately classified by the model. Net benefit was maximized with threshold probabilities of 0%-19% by the “3D-ADC-DWI-pp-T2” model. If the threshold probability was more than 19%, the “3D-ADC-ap-DWI-T2” model would add more net benefit compared with the other radiomics models across the majority of the range of threshold probabilities.

## Discussion

The results of our study suggested that radiomics models using different MRI-based multisequence TA has the potential for identifying the TP53 mutation status in PDAC.

We extracted several quantitative 2D and 3D radiomics features from seven different MRI sequences and constructed 378 SVM classifiers, including 2D,3D and the combined 2D and 3D modalities. Subsequently, three models with better predictive performance were selected to predict TP53 status in pancreatic cancer. The candidate features were reduced to 11 potential variables using the DX score, based on the accuracy of the SVM classifiers. SVM and DX scores were used at the same time, achieving a good classification accuracy of the TP53 mutation status in PDAC patients. All features extracted from 3D in consist with the previous study,3D analysis may provide a more representative evaluation of tumor heterogeneity (23). Although no independent validation cohort was available, the model was validated using five-fold cross-validation, which is a common procedure for model validation with a limited number of samples. Here, the combined model proved valid in the cross-validation yielding high diagnostic accuracies above 91.0%.

Radiomics have been previously correlated with a specific genotype or molecular phenotype in different cancer types such as lung (24), brain (25), and rectal cancer (26). However, radiomics had previously seen limited use in PDAC characterization. Marc A. Attiyeh et al. (27) found an association between resectable PDAC imaging features and SMAD4 status using CT texture analysis, but the model did not predict TP53 status. Si Shi et al. (28) found a correlation of PET-imaging features with TP53 status in terms of metabolic tumour burden. Yosuke et al. had reported a model for predicting TP53 mutations in pancreatic cancer from CT images using machine learning (29), and its AUC value was 0.795. But CT had no high contrast resolution that can reflect indistinguishable lesions in PDAC. Our results found that 3D texture features from T2WI, ADC, DWI and DCE T1WI in ap were influential in the analysis to find a classifier for TP53 status characterization.

SGT-53 is a gene therapy anti-cancer therapeutic agent comprised of a cationic liposome encapsulating a plasmid encoding wild-type p53. A phase I trial showed that SGT-53 is well tolerated, exhibits anticancer activity and reaches metastatic lesions in patients with different solid tumors (30). And a Phase II clinical trial of SGT-53 plus gemcitabine and nab-paclitaxel (NCT02340117) was used for metastatic pancreatic cancer. In the future, TP53 status in PDAC may play a greater role in treatment selection.

There are several limitations in our study. First, this was a retrospective study from a single center and population was small. More data from multiple institutions were needed to validate our results and acquired the optimized model. Moreover, the effect of field strength (1.5 T and 3 T) on radiomics had been unclear due to the limitation of the retrospective data. Thus, we used normalization of the values extracted from images to improve the repeatability of features. Second, the method of including patients who underwent surgery would also lead to selection bias. Finally, we used only one software for the texture analysis and one method. Therefore, the applicability of our model to other software and algorithms is uncertain.

## Conclusion

In conclusion, the radiomics model derived from mp-MRI provided a non-invasive, quantitative method to predict TP53 mutation status in PDAC. Therefore, this radiomics model may help clinicians to select optimal therapies in patients with PDAC.

## Data Availability Statement

The original contributions presented in the study are included in the article/**Supplementary Material**. Further inquiries can be directed to the corresponding authors.

## Ethics Statement

The studies involving human participants were reviewed and approved by Institutional Review Board of Ruijin Hospital affiliated to Shanghai Jiao Tong University School of Medicine. The ethics committee waived the requirement of written informed consent for participation.

## Author Contributions

Study concept and design: XzL and XhQ. Acquisition and segmentation of image: JG, XdL, and WhF. Analysis and interpretation of data: XhC. Clinical studies: BL and FM. Draft of the initial manuscript: JG and XhC. Further review and editing of the manuscript: XzL and XhQ. All authors contributed to the article and approved the submitted version.

## Funding

This study was supported by research grants from the National Natural Science Foundation of China (62073218), Shanghai Science and Technology Committee of Shanghai Municipality (20Y11912300), and Shanghai Municipal Key Clinical Specialty (shslczdzk03403).

## Conflict of Interest

The authors declare that the research was conducted in the absence of any commercial or financial relationships that could be construed as a potential conflict of interest.
