# Supplementary material for: Differentiating TP53 Mutation Status in Pancreatic Ductal Adenocarcinoma Using Multiparametric MRI-Derived Radiomics
Source: Front Oncol. 2021 May 17;11:632130. doi: 10.3389/fonc.2021.632130 (PMC8165316; doi:10.3389/fonc.2021.632130)
Supplement: Supplementary file 1 [file Table_1.docx]

**Supplementary information**

***for***

**Differentiating TP53 mutation status in pancreatic ductal adenocarcinoma using MRI-derived radiomics**

Number of tables: 2

**Table 1.** MRI sequence parameters

| Image | Slice thickness(mm) | | TR (ms) | | TE (ms) | | FA (°) | |
| --- | --- | --- | --- | --- | --- | --- | --- | --- |
|  | 1.5T | 3.0T | 1.5T | 3.0T | 1.5T | 3.0T | 1.5T | 3.0T |
| T1WI+fs | 5.0 | 2.0 | 215 | 3.7 | 2.16 | 1.32 | 80 | 10 |
| T2WI+fs | 5.0 | 6.0 | 6000 | 2750 | 85.0 | 77.59 | 90 | 90 |
| DWI | 5.0 | 6.0 | 7500 | 1383 | 67.5 | 55.18 | 90 | 90 |
| DCET1+fs | 4.4 | 2.5 | 3.85 | 3.2 | 1.83 | 1.50 | 15 | 10 |

T1WI+fs, precontrast T1-weighted imaging with fat-suppression

T2WI+fs, T2-weighted imaging with fat-suppression

DWI, diffusion-weighted imaging

DCE T1WI+fs, dynamic contrast-enhanced T1-weighted imaging with fat-suppression

TR: repetition time

TE: echo time

FA:flip angle

**Table 2.** The category and number of features

|  |  |  | **shape** | **First Order feature** | **GLCM**  **Feature** | **GLDM Feature** | **GLRLM Feature** | **GLSZM**  **Feature** | **NGTDM**  **Feature** | **Total** |
| --- | --- | --- | --- | --- | --- | --- | --- | --- | --- | --- |
| 2D  features | Original image | |  | 18 | 24 | 14 | 16 | 16 | 5 | 93 |
|  | Wavelet  Transformed  images | LH |  | 18 | 24 | 14 | 16 | 16 | 5 | 93 |
|  |  | HL |  | 18 | 24 | 14 | 16 | 16 | 5 | 93 |
|  |  | HH |  | 18 | 24 | 14 | 16 | 16 | 5 | 93 |
|  |  | LL |  | 18 | 24 | 14 | 16 | 16 | 5 | 93 |
|  | gradient images | |  | 18 | 24 | 14 | 16 | 16 | 5 | 93 |
|  |  |  |  |  |  |  |  |  |  | 558 |
| 3D features | Original images | | 14 | 18 | 24 | 14 | 16 | 16 | 5 | 107 |
|  | wavelet transformed  images | LLH |  | 18 | 24 | 14 | 16 | 16 | 5 | 93 |
|  |  | LHL |  | 18 | 24 | 14 | 16 | 16 | 5 | 93 |
|  |  | LHH |  | 18 | 24 | 14 | 16 | 16 | 5 | 93 |
|  |  | HLL |  | 18 | 24 | 14 | 16 | 16 | 5 | 93 |
|  |  | HLH |  | 18 | 24 | 14 | 16 | 16 | 5 | 93 |
|  |  | HHL |  | 18 | 24 | 14 | 16 | 16 | 5 | 93 |
|  |  | HHH |  | 18 | 24 | 14 | 16 | 16 | 5 | 93 |
|  |  | LLL |  | 18 | 24 | 14 | 16 | 16 | 5 | 93 |
|  | gradient images | |  | 18 | 24 | 14 | 16 | 16 | 5 | 93 |
|  |  |  |  |  |  |  |  |  |  | 944 |

GLCM: grey-level co-occurrence matrix

GLDM: gray level dependence matrix

GLRLM: grey-level run-length matrix

GLSZM: gray level size zone matrix

NGTDM: neighbouring gray-tone difference matrix
